# Study on the Biomolecular Competitive Mechanism of Polybrominated Diphenyl Ethers and Their Derivatives on Thyroid Hormones

**DOI:** 10.3390/molecules28217374

**Published:** 2023-10-31

**Authors:** Shaoheng Liu, Rong Hu, Hao Zhan, Wanli You, Jianjun Tao, Luhua Jiang

**Affiliations:** 1 Engineering Research Center of Hunan Province for Recycling Technology of Electroplating Wastewater, Hunan Provincial Key Laboratory of Water Treatment Functional Materials, College of Chemistry and Material Engineering, Hunan University of Arts and Science, Changde 415000, China; 2 College of Environment, South China Normal University, Guangzhou 510006, China; 3 College of Environmental Science and Engineering, Guilin University of Technology, Guilin 541006, China; 4 Key Laboratory of Biometallurgy of Ministry of Education, College of Minerals Processing and Bioengineering, Central South University, Changsha 410083, China

**Keywords:** PBDEs, OH-PBDEs, thyroid interference, fluorescence probe, molecular docking

## Abstract

Polybrominated diphenyl ethers (PBDEs) are widely used brominated flame retardants. PBDEs and their derivatives, hydroxylated PBDEs (OH-PBDEs), can bind to hormone receptors and impact hormone secretion, transportation, and metabolism, leading to endocrine disruption and the development of various diseases. They have particularly strong interference effects on thyroid hormones. This study used decabromodiphenyl ether (BDE-209); 2,2′,4,4′-tetrabromodiphenyl ether (BDE-47); and 6-OH-BDE-47 as representative compounds of PBDEs and their derivatives, OH-PBDEs. A fluorescence probe, fluorescein-isothiocyanate-L-thyroxine (FITC-T4, F-T4), specific for binding to transthyretin (TTR), a thyroid transport protein, was prepared. The binding capacity of PBDEs and their derivatives, OH-PBDEs, to TTR was quantitatively measured using fluorescence spectroscopy. The principle of quenching the fluorescence intensity of F-T4 after binding to TTR was used to analyze the competitive interaction between the probe and BDE-209, BDE-47, and 6-OH-BDE-47, thereby evaluating the toxic effects of PBDEs and their derivatives on the thyroid system. Additionally, AutoDock molecular docking software (1.5.6) was used to further analyze the interference mechanism of OH-PBDEs on T4. The results of the study are as follows: (1) Different types of PBDEs and OH-PBDEs exhibit varying degrees of interference with T4. Both the degree of bromination and hydroxylation affect their ability to competitively bind to TTR. Higher bromination and hydroxylation degrees result in stronger competitive substitution. (2) The competitive substitution ability of the same disruptor varies at different concentrations. Higher concentrations lead to stronger substitution ability, but there is a threshold beyond which the substitution ability no longer increases. (3) When OH-PBDEs have four or more bromine atoms and exhibit the most structural similarity to T4, their binding affinity to TTR is stronger than that of T4.

## 1. Introduction

Persistent organic pollutants (POPs) have received significant international attention due to their long-term persistence, bioaccumulative nature, semi-volatility, and high toxicity. Polybrominated diphenyl ethers (PBDEs), as a new type of POPs, were listed in the Stockholm Convention on Persistent Organic Pollutants in 2009 [1]. The production and use of pentabrominated and octabrominated diphenyl ethers are globally banned. PBDEs are a class of brominated compounds with excellent flame retardant properties, widely used in textile, chemical, electronic products, furniture, and construction materials [2]. There are a total of 209 congeners of PBDEs. Among them, decabromodiphenyl ether (BDE-209) has the lowest acute toxicity and is the most widely used flame retardant, accounting for over 75% of the total global production [3]. According to statistics, China alone produced 30,000 tons of BDE-209 in 2005 [4]. Due to its large-scale usage, the accumulation concentration of BDE-209 in environmental media has rapidly increased. It was also added to the list of newly controlled POPs in 2017. Since PBDEs are physically added to flame retardants rather than through chemical bonds, they can be easily released into the environment during the production, use, and recycling processes.

**Figure 1:**
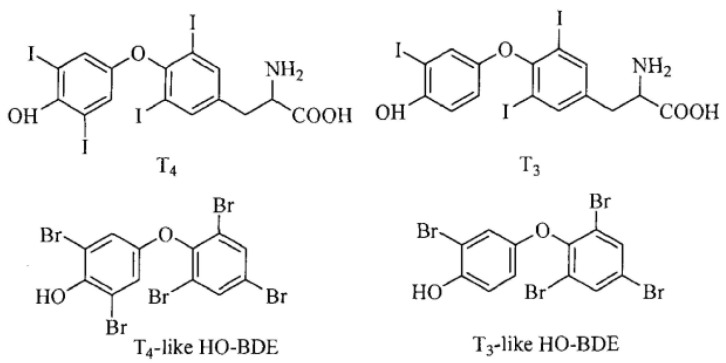
Chemical structures of T4, T3, and OH-PBDEs similar to them.

PBDEs have stable chemical properties, hydrophobicity, low volatility, and bioaccumulation potential [5]. They have potential carcinogenic effects on the human body and toxic effects on the nervous, endocrine, and immune systems [6,7]. The metabolites of PBDEs, hydroxylated polybrominated diphenyl ethers (OH-PBDEs), have even stronger hydrophobicity and toxicity. Studies have shown that PBDEs can bind to hormone receptors and affect hormone secretion, transport, and metabolism, causing the disruption of the endocrine system and leading to the development of various diseases [8,9]. The environmental endocrine disruption effects of PBDEs mainly manifest in the interference with thyroid hormones. Currently, most studies on the effects of PBDEs on thyroid function focus on their impact on thyroid hormone levels, such as triiodothyronine (T3), thyroxine (T4), and thyroid-stimulating hormone (TSH) [10]. According to the study of Klaren [11], the biological of thyroid hormone levels are mainly determined by deiodinase. PBDEs can exert toxic effects on deiodinases and affect its concentration. Treatment with different concentrations of PBDEs significantly affects the transcription levels and gene expression of deiodinases. The mechanisms of PBDEs’ effects on thyroid hormones are not fully understood, but two possible mechanisms are generally believed to exist. Firstly, PBDEs can induce changes in the activity of various enzymes involved in thyroid hormone metabolism, including CYPIA1, CYP2B, and UDPGT [12,13]. These enzymes play important roles in the metabolism of thyroid hormones. Secondly, PBDEs have a structural similarity to T3 and T4, and the metabolism of PBDEs can also generate hydroxylated diphenyl ethers that are structurally more similar to thyroid hormones. OH-PBDEs, as well as T3 and T4, all have a basic structure composed of biphenyl ethers, and they all have halogen and hydroxyl substitutions on the benzene ring (as shown in Figure 1). This structural similarity allows PBDEs to interfere with the transport of thyroid hormones [14,15]. Currently, most research on the environmental endocrine disruption effects of PBDEs is based on cell or animal experiments, and the molecular-level mechanism studies are relatively limited, lacking the mutual verification of results from different perspectives of research [16,17].

**Figure 2:**
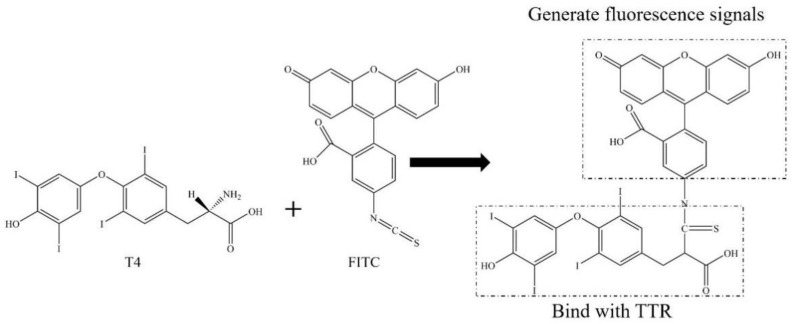
Schematic diagram of F-T4 synthesis.

By preparing the fluorescein isothiocyanate-L-thyroxine fluorescence probe (FITC-T4, F-T4) capable of specifically binding to transthyretin (TTR), the binding capacity of PBDEs and their derivatives, OH-PBDEs, with TTR can be quantitatively determined using fluorescence spectroscopy. The schematic diagram of F-T4 synthesis is presented in Figure 2. It can be observed that in this reaction, the free amino group in the T4 molecule acted as a nucleophile and underwent nucleophilic addition reaction with the carbon atom on the isothiocyanate group of the FITC molecule, resulting in the synthesis of F-T4 where T4 and FITC are linked together. The T4 portion of this molecule is used to bind to the thyroid hormone receptor protein TTR, while FITC provides the fluorescent signal for detection. The FITC-T4 probe is labeled with a fluorescein group on the T4 molecule. After labeling, the iodine atom in the T4 molecule is close to the amino group of FITC, which leads to the quenching of FITC fluorescence due to collision and heavy atom effects. However, when TTR binds to F-T4, the iodine atom of T4 enters the internal structure of TTR, causing it to move away from the fluorescein group FITC and restore the fluorescence intensity of FITC [18]. In this case, if the thyroid disruptors PBDEs or OH-PBDEs are present and compete with T4 for TTR, some of the T4 will be displaced from the binding site with TTR, exposing the iodine atom of T4 again and causing it to bind to FITC, resulting in a decrease in FITC fluorescence intensity [19]. The strength of the competition effect of thyroid disruptors can be reflected by the fluorescence intensity of F-T4. Therefore, the principle of fluorescence quenching of F-T4 due to the binding of the disruptors to TTR can be used to analyze the competition of this probe with PBDEs and OH-PBDEs.

The molecular docking theory originated from Fisher’s “Lock and Key” theory and is now widely applied in various fields, such as pharmacy, medicine, environmental science, and agricultural science. The molecular docking method can simulate the interaction between a small molecule ligand (such as T4 or its disruptors) and a protein receptor (such as TTR) at the atomic level, predicting the conformation and binding position of the ligand, evaluating the binding affinity between the ligand and the receptor, and describing the performance of the small molecule on the binding site of the target protein, elucidating the binding mechanism of the ligand-receptor. Through computational simulations using molecular docking, not only can the economic costs of experimental studies be reduced, but also the basic characteristics of the binding site can be quickly revealed at the molecular level. On one hand, the molecular docking theory is used for predicting the properties of various compounds, and on the other hand, it can also be used to explain experimental phenomena and elucidate the underlying mechanisms of interactions. Therefore, Autodock molecular docking software (1.5.6) can be used to analyze the binding affinity of OH-PBDEs and T4 with TTR to clarify the mechanisms of interference of OH-PBDEs with T4.

In this study, polybrominated diphenyl ether 209 (BDE-209); 2,2′,4,4′-tetrabromodiphenyl ether (BDE-47); and 6-OH-BDE-47 were selected as representative substances of high-molecular-weight PBDEs, low-molecular-weight PBDEs, and their derivative, OH-PBDEs, respectively. The combined use of an fluorescence probe assay and the molecular docking method was employed to verify the interference of PBDEs and OH-PBDEs with T4 binding to TTR at different perspectives, in order to evaluate the toxic effects of PBDEs and their derivatives on the thyroid system.

## 2. Results and Discussion

### 2.1. Synthesis, Purification, and Optimization of F-T4 Fluorescent Probe

**Figure 3:**
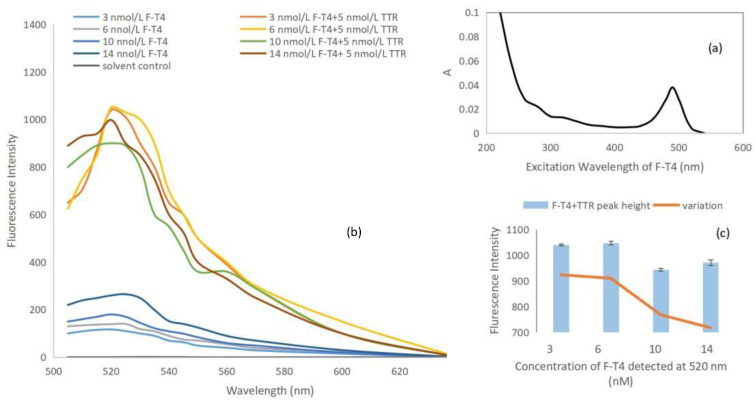
Synthesis and purification of F-T4 (**a**), optimization (**b**), peak height and change value of fluorescence curve before and after combination of F-T4 with TTR at 520 nm (**c**).

The purified orange-red F-T4 fluorescent probe was scanned using a UV-Vis spectrophotometer in the wavelength range of 200–700 nm to detect its excitation wavelength. The results showed a unique distinct maximum absorption peak at approximately 490 nm [20], as shown in Figure 3a. Based on previous literature, the excitation wavelength of F-T4 probe was determined to be 490 nm [20], indicating that the fluorescent probe was synthesized successfully. Based on the orange–red color of the probe, and the clear and pure absorption peak observed in the UV scanning spectrum, it can be determined that the probe has a relatively high purity.

Under excitation at 490 nm, the fluorescence intensity of different concentrations of probes was measured after adding 5 nmol/L of TTR using a fluorescence spectrometer. As shown in Figure 3b, F-T4 exhibited strong fluorescence at around 520 nm, indicating that its emission wavelength is 520 nm. Moreover, when TTR was bound to different concentrations of F-T4, the fluorescence intensity increased due to the iodine atom on T4 moving away from the fluorophore. This demonstrated a certain concentration-dependent relationship and further confirmed the successful synthesis and purification of the fluorescence probe. The maximum fluorescence intensity was observed when the concentration of TTR was 5 nmol/L and the concentration of F-T4 was 1.6 × 10^−5^ (approximately 6 nmol/L) of the stock solution (Figure 3c). The fluorescence intensity was 1047.33, with a fluorescence intensity change of 909.86. This value was slightly lower than the maximum fluorescence intensity change 923.64 of 3 nmol/L F-T4, but the emission wavelength remained stable. Therefore, a concentration of 6 nmol/L F-T4 was chosen for subsequent competition experiments.

### 2.2. Binding Competition of PBDEs and OH-PBDEs with F-T4 and TTR

**Figure 4:**
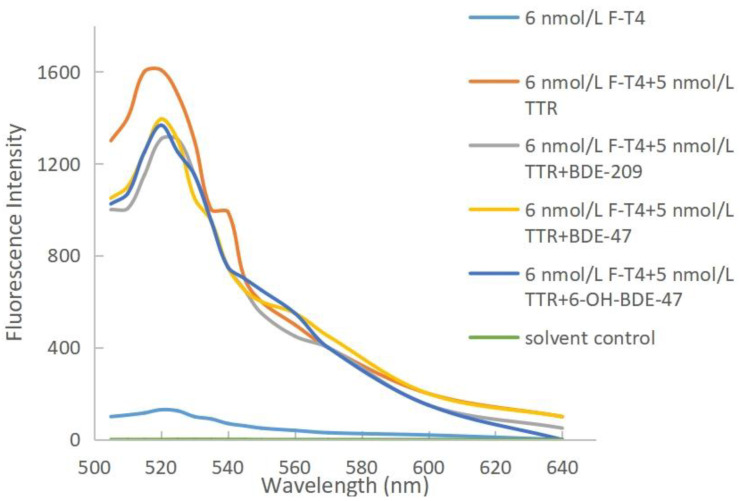
TTR fluorescence spectra of PBDEs and OH-PBDEs competing with T4.

The thyroid-disrupting effects of PBDEs and OH-PBDEs can be reflected by the magnitude of fluorescence quenching. The stronger the disruptive effect, the more binding with TTR, leading to more displacement of free F-T4 and a higher fluorescence-quenching intensity. Fluorescence quenching can be caused by energy transfer, complex formation, collision, or excited state reactions [21]. The mechanisms of aromatic fluorescence quenching include dynamic quenching and static quenching. Dynamic quenching occurs through interactions between the excited-state molecules of the fluorophore and the quencher, and is often positively correlated with reaction temperature [22]. On the other hand, static quenching involves reactions between the ground-state molecules of the fluorophore and the quencher, and the quenching constant decreases with increasing reaction temperature [23,24]. The results of the study are shown in Figure 4. The fluorescence intensity of the experimental groups decreased compared to the F-T4 and TTR system alone, indicating that all three substances, BDE-209, BDE-47, and 6-OH-BDE-47, were able to displace F-T4 from TTR. PBDEs and OH-PBDEs exhibit a certain degree of thyroid disrupting effect.

The thyroid disrupting intensities of these three substances vary, which is related to the degree of bromination and hydroxylation [25]. When the concentrations of BDE-209, BDE-47, and 6-OH-BDE-47 were at their maximum levels of 1 μmol/L, the resulting decrease in fluorescence intensity was 300, 213, and 240, respectively. Among the three interfering substances, BDE-209 has the highest degree of bromination, and it also shows the largest decrease in fluorescence intensity. This indicates that BDE-209 is more capable of replacing T4 from TTR, suggesting that the degree of bromination of interferents affects the binding ability between T4 and TTR, with higher bromination leading to stronger substitution ability. In comparison, 6-OH-BDE-47 and BDE-47 have an equal number of bromine atoms, but 6-OH-BDE-47 exhibits a slightly larger decrease in fluorescence compared to BDE-47. This suggests that the presence of the hydroxyl group of interferents also affects the binding ability between T4 and TTR.

**Figure 5:**
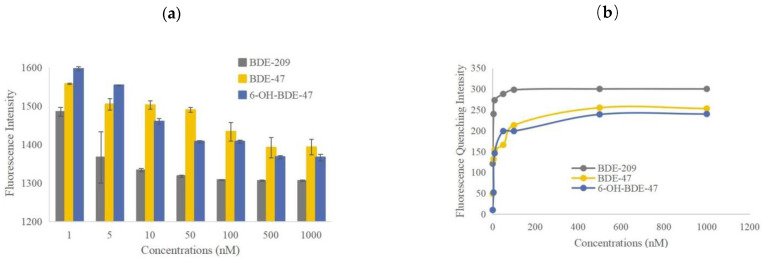
Fluorescence intensity (**a**) and fluorescence quenching intensity (**b**) of TTR in competition with different concentrations of thyroid disruptors (PBDEs and OH-PBDEs) as compared to T4.

The fluorescence intensity (a) and fluorescence quenching intensity (b) of TTR in competition with different concentrations of thyroid disruptors at the maximum emission wavelength of 520 nm are shown in Figure 5. According to Figure 5a, as the concentrations of BDE-209, BDE-47, and 6-OH-BDE-47 gradually increased from 1 × 10^−9^ to 1 × 10^−6^ mol/L, the fluorescence intensity of the three systems decreased progressively. The fluorescence intensity decreased from 1485 to 1306, 1558 to 1394, and 1597 to 1366, respectively. The fluorescence quenching intensity increased from 121 to 300, 49 to 213, and 10 to 240, respectively (Figure 5b).

The fluorescence quenching phenomenon occurred in all three systems, indicating that the three interfering substances caused the partial displacement of T4 from the binding sites with TTR, resulting in free T4. This exposed the iodine atoms in T4, leading to their binding with FITC and subsequently reducing the fluorescence intensity of FITC. This further confirms that the interference ability of PBDEs and OH-PBDEs varies depending on the number of bromine atoms and the presence of hydroxyl groups. The higher the degree of substitution, the stronger the interference, resulting in a higher amount of free F-T4 and a higher efficiency of fluorescence quenching. Additionally, the interference effect also varies with the concentration of the interfering substances. As the concentration of thyroid disruptors increased, the competitive displacement also became stronger. However, when the concentrations of these three disruptors increased to 1 × 10^−7^, 0.5 × 10^−6^, and 0.5 × 10^−6^ mol/L, respectively, the change in fluorescence intensity is small, and the efficiency of fluorescence quenching has reached a plateau. This means that when the concentration of the interfering substance reaches a certain upper limit, the competition for displacement by the interfering substance approaches saturation due to the limited number of binding sites between the small molecule and TTR.

### 2.3. Binding Affinities of OH-PBDEs and T4 to TTR

The binding affinity reflects the binding capability between a ligand and a large molecular protein. The larger the absolute value of the binding affinity, the better the binding ability [26]. When two ligands compete for the same binding site, the ligand with higher binding affinity is more likely to bind to the receptor protein [27,28]. Therefore, comparing the binding affinities of OH-PBDEs and T4 to TTR can further elucidate the thyroid disruption mechanism of OH-PBDEs. Due to the large size of the TTR macromolecule, there are many sites within it that can bind to T4 molecules. However, there is only one specific site where the docking results in the tightest binding, resulting in the highest absolute value of binding affinity.

**Figure 6:**
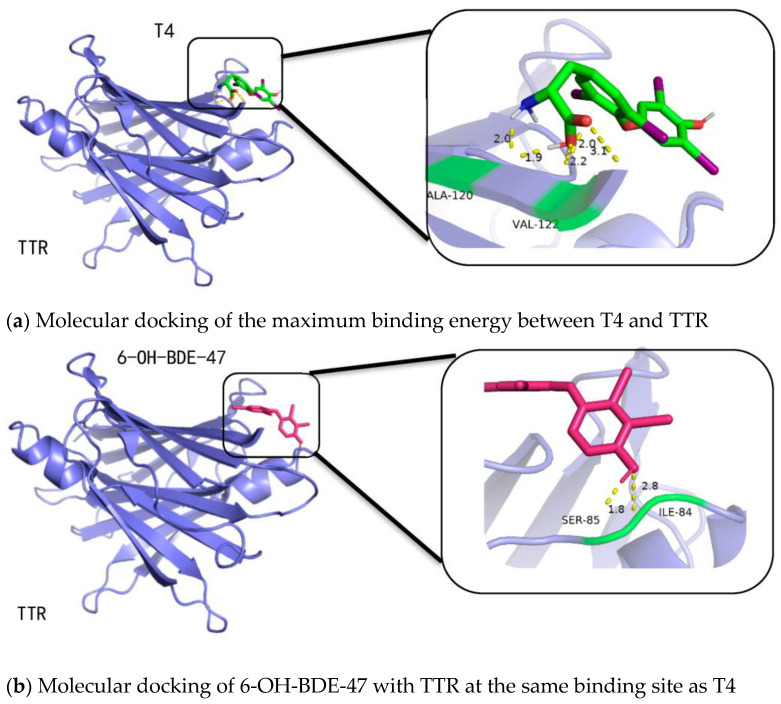

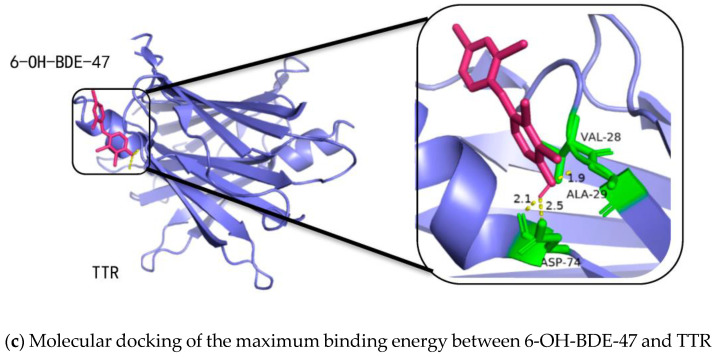
Molecular docking of T4 (**a**) and 6-OH-BDE-47(**b**,**c**) with TTR.

Figure 6a shows the binding of TTR with T4 at the site allowing for maximum binding energy, where T4 is connected to the residues of alanine and valine on TTR through hydrogen bonding. Taking 6-OH-BDE-47 as an example, comparing Figure 6a,b, it can be observed that at the same binding pocket as T4, the interfering substance tends to bind to the residues of isoleucine and serine. The binding energy is −5.16 kcal/mol, which is lower than the binding energy of T4 with TTR (−6.63 kcal/mol), indicating a certain level of interference. Comparing Figure 6a,c, it can be observed that the interfering substance 6-OH-BDE-47 has a different optimal binding site with TTR compared to T4. In this case, 6-OH-BDE-47 is connected to the residues of alanine and aspartic acid on TTR through hydrogen bonding. The binding affinity is −6.49 kcal/mol. The optimal binding site of 6-OH-BDE-47 is on the inner side of TTR, while the optimal binding site of T4 is on the outer side of TTR. The main reason for this difference may be that 6-OH-BDE-47 belongs to low-brominated disruptors with a smaller molecular volume, allowing it to fit inside the binding pocket. It forms stable binding through hydrogen bonding between its hydroxyl group and the amino acid residues of the protein side chain, as well as hydrophobic interactions between the bromine atoms on the benzene ring and the amino acid residues of the side chain.

Therefore, from Figure 6a–c, it can be observed that the optimal binding affinity of the interfering substance 6-OH-BDE-47 is slightly lower than that of T4 with TTR, but it still exhibits strong thyroid-disrupting effects. This may be due to the fact that when interfering substances like OH-PBDEs bind to TTR, they induce conformational changes on the protein surface, which affects the binding affinity between T4 and TTR.

**Figure 7:**
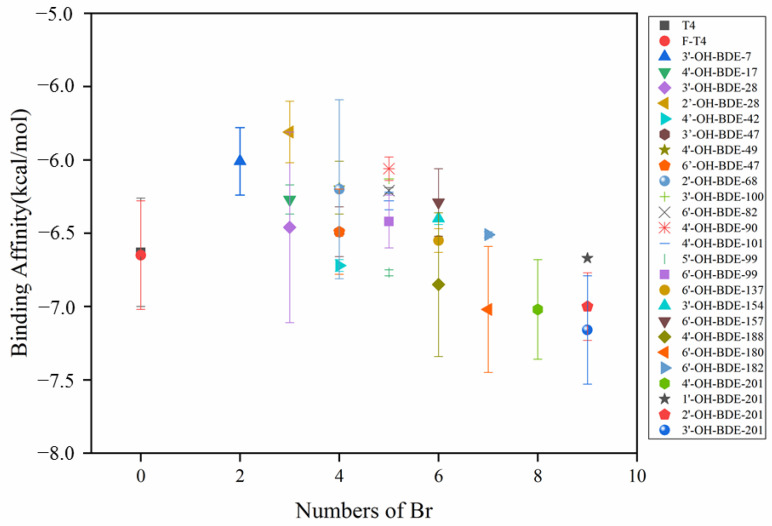
The distribution of the interference effects based on the number of bromine atoms in OH-PBDEs and T4.

This study analyzed the binding affinities of 25 different OH-PBDEs with 1–9 bromine atoms to TTR, as shown in Figure 7 and Table S1 (Supplementary Materials). The number of bromine atoms in the interfering substances and the position of hydroxyl substitutions in the molecule can affect their disruptive effects on T4. Generally, the binding strength between TTR and the natural hormone T4 is greater than that of the interfering substances, and the disruptive effect increases with the increasing number of bromine atoms. When the number of bromine atoms in OH-PBDEs is four or more and the position of hydroxyl groups in the molecule is most similar to that in T4, the binding affinity with TTR is stronger than that with T4, resulting in a stronger disruptive effect.

## 3. Materials and Methods

### 3.1. Materials

BDE-209, BDE-47, FITC, TTR, pyridine, triethylamine, glacial acetic acid and Tris were obtained from Shanghai Maclin Biochemical Technology Co., Ltd. (Shanghai, China). 6-OH-BDE-47 (50 μg/mL in nonane) was obtained from Annoron (Beijing, China) Biochemical Technology Co., Ltd. T4 was obtained from Beijing Biolab Technology Co., Ltd (Beijing, China). Sephadex G-75 was obtained from Shanghai Yuanye Biochemical Technology Co., Ltd. (Shanghai, China). Others were obtained from Tianjin Damao chemical reagent factory (Tianjin, China).

### 3.2. Preparation and Synthesis of F-T4 Fluorescent Probe

Based on previous studies [20], F-T4 fluorescent probe was prepared for this research. The specific method is as follows: FITC and T4 were dissolved in a mixed solvent of triethylamine/water/pyridine (volume ratio of 0.1:1.5:9) to prepare solutions of FITC and T4 with a concentration of 20 mg/mL each. In a beaker, 10 mL of 20 mg/mL FITC and 20 mL of 20 mg/mL T4 were added and reacted at room temperature for 1 h. After the reaction, 9 mL of the resulting mixture was added to a 60 mL solution of 0.2 mol/L ammonium acetate buffer with a pH of 4.0 to precipitate the mixture. If there is no obvious change in the solution, a small amount of ice acetic acid can be added multiple times to promote the precipitation of the orange precipitate. After high-speed centrifugation for 15 min, the supernatant was discarded and the precipitate was retained. The precipitate was washed twice with ultrapure water by centrifugation. Then, 15 mL of 0.05 mol/L ammonium bicarbonate was added to redissolve the precipitate. When there was no significant change in the content of the precipitate, 5 mL of ammonia was added to quickly dissolve the precipitate, resulting in an orange-red mixture solution, which is the purified F-T4 fluorescent probe.

### 3.3. Purification and Storage of F-T4 Fluorescent Probe

5 g of sephadex gel G-75 was swollen in 100 mL of a 0.5 mol/L ammonium bicarbonate solution for 48 h, and then packed into a chromatography column with dimensions of 3 × 30 cm. The column was equilibrated with 0.5 mol/L ammonium bicarbonate, and the sample to be purified was added in one go. Before purification, a 0.5 mol/L ammonium bicarbonate solution was used as an unfolding agent. The prepared F-T4 mixture was aspirated with a capillary tube and spotted on a silica gel plate. The plate was then observed under UV light with a wavelength of 365 nm to observe the migration pattern of the mixture. Due to the difference in molecular weight and polarity between free FITC and F-T4, free FITC has a higher solubility in the unfolding agent, resulting in a faster migration speed and appearing at the lower end of the plate as a light-yellow color. On the other hand, F-T4 appears as an orange-red color with a slower migration speed, appearing at the upper end of the plate. The purification was performed using 0.5 mol/L ammonium bicarbonate as the elution solution to separate the free FITC and the F-T4 fluorescent probe. The purified orange-red F-T4 fluorescent probe was stored as a stock solution (1) at −20 °C or lyophilized into powder and stored at 4 °C for future use.

### 3.4. Binding of F-T4 Fluorescent Probe with TTR

In order to obtain a better representation of fluorescence intensity, different concentrations of the fluorescent probe F-T4 were used in binding experiments with TTR. The concentration of F-T4 that showed the highest change in fluorescence intensity was selected as the optimal concentration for the competition-binding experiments.

The purified stock solution (1) of F-T4 fluorescent probe was diluted to 8 × 10^−5^ times of the original concentration to prepare the F-T4 stock solution (2). A stock solution (3) of 10 nmol/L TTR was prepared and stored at −20 °C. Different volumes of the stock solutions (2) and (3) were added to a Tris-Nacl buffer solution (50 mM Tris-HCl/100 mM NaCl, pH = 7.4) while maintaining a total volume of 1 mL, resulting in a TTR concentration of 5 nmol/L and F-T4 concentrations of 8 × 10^−6^, 1.6 × 10^−5^, 2.5 × 10^−6^, and 3.3 × 10^−5^ times of stock (1), respectively, which was 3, 6, 10, 14 nmol/L, respectively. A blank control group without TTR was used as a control. After incubating at room temperature for 30 min, 100 μL of the reaction mixture from each experimental group was transferred to a quartz cuvette and diluted to a final volume of 1 mL with detection solution. The fluorescence intensity was measured using a fluorescence spectrophotometer. Three parallel experiments were performed for each group.

The stock solution (1) of F-T4 fluorescent probe was diluted to a concentration of 10^−4^, and the absorbance spectrum was scanned in the wavelength range of 200–700 nm using a UV-visible spectrophotometer (Shimadzu UV-2600) to determine the maximum absorption wavelength at 490 nm, which was used as the excitation wavelength (λ_ex_) for fluorescence detection. The F-T4 concentration was roughly determined based on its UV absorption at 490 nm, with a molar absorptivity of approximately 7.8 × 104 M^−1^ cm^−1^ [20]. The emission wavelength was scanned in the range of 505–700 nm using a fluorescence spectrophotometer (HITACHI, F-7000), and a significant absorption peak was observed at 520 nm, which was determined as the emission wavelength (λ_em_). Each point of an emission spectrum was measured three times.

### 3.5. Competition between F-T4 and BDE-209, BDE-47, and 6-OH-BDE-47

To investigate the competitive binding of PBDEs and OH-PBDEs with T4 to TTR, a gradient concentration analysis was performed for BDE-209, BDE-47, and 6-OH-BDE-47, to determine their binding abilities with TTR.

The F-T4 concentration with the maximum change in fluorescence intensity from step 1.4 was selected, and gradient concentrations of BDE-209, BDE-47, and 6-OH-BDE-47 (all diluted in acetonitrile) at 1 × 10^−9^, 5 × 10^−9^, 1 × 10^−8^, 0.5 × 10^−7^, 1 × 10^−7^, 0.5 × 10^−6^, and 1 × 10^−6^ mol/L were mixed with TTR (5 nmol/L) in Tris-Nacl buffer solution (50 mM Tris-HCl/100 mM NaCl, pH = 7.4) at room temperature for 30 min. The total volume of the reaction mixture for each system was 100 μL. Subsequently, 100 μL of the reaction mixture from each experimental group was transferred to a quartz cuvette and diluted to a final volume of 1 mL with detection solution. The fluorescence intensity was then measured at 520 nm. All experiments were conducted three times.

### 3.6. Determination of Binding Affinity of 6-OH-PBDEs

It is difficult to meet the requirements of ecological risk assessment of organic pollutants due to the significant material, manpower, and time costs involved in the method of combining each interference substance’s small molecule with a fluorescent probe and then comparing its competitive effect with T4. However, molecular docking can provide more information on the interaction between ligands (T4 or its interference substances) and the receptor TTR, which is beneficial for a deeper understanding of the molecular interactions. In order to validate the thyroid-disrupting effects of PBDEs and their derivatives, OH-PBDEs, from different perspectives, this study utilized molecular docking. The molecular structure of TTR was downloaded from the PDB database (PDB DOI: 10.2210/pdb1DVQ/pdb, accessed on 7 December 2011) as the receptor protein. Using Avogadro software (1.2.0n), 25 different OH-PBDEs with 1–9 bromine atoms and the T4 small molecule structure were constructed. AutoDock molecular docking software was used to analyze the molecular docking of TTR with OH-PBDEs and T4. The TTR protein is set as a rigid structure, while the ligand small molecule is considered flexible and rotatable. When using AutoGrid for calculations, The grid box (size = 47.25 × 47.25 × 47.25 Å) was centered on TTR to find the suitable space for ligand binding. The distance between the grid points of the box was set to 0.375 Å. The interference mechanism of OH-PBDEs was determined based on the strength of the docking binding. The molecular docking experiments were conducted in triplicate.

## 4. Conclusions

PBDEs are a class of brominated compounds known for their excellent flame retardant properties and are widely used in the production of textiles, chemicals, electronics, furniture, and building materials. The environmental exposure to PBDEs and their derivatives, OH-PBDEs, carries the potential for the occurrence of various disorders associated with thyroid dysfunction. This study prepared F-T4 fluorescent probes and utilized the principle that the binding of disruptors to TTR leads to quenching of F-T4 fluorescence intensity. Combined with molecular docking experiments, the binding affinities of OH-PBDEs and TTR were determined, and the thyroid disruption effects of PBDEs and their derivatives, OH-PBDEs, were analyzed at the molecular level from different perspectives. The conclusions are as follows:

(1) PBDEs and OH-PBDEs have certain thyroid interference effects. Different types of interferents having different effects. The binding capacity of PBDEs and OH-PBDEs to TTR is correlated with the number of bromine atoms in the PBDEs. The more bromine atoms in the molecule, the stronger the binding affinity with TTR.
(2) The binding affinity of PBDEs and their derivatives to TTR is related to the presence of hydroxyl functional groups. When the number of bromine atoms is the same, OH-PBDEs exhibit stronger binding affinity to TTR than PBDEs.
(3) The binding affinity of PBDEs and OH-PBDEs to TTR is influenced by the concentration of the disruptors. Higher concentrations result in stronger competition between the disruptors and F-T4 for binding to TTR. When the concentrations of BDE-209, BDE-47, and 6-OH-BDE-47 increased from 1 nmol/L to 1 μmol/L, the changes in fluorescence quenching of F-T4 upon competition with TTR increase from 121 to 300, 49 to 213, and 10 to 240, respectively.
(4) When OH-PBDEs have four or more bromine atoms and exhibit the most structural similarity to T4, their binding affinity to TTR is stronger than that of T4.

## Data Availability

The study did not report any data.

## Supplementary Materials

The following supporting information can be downloaded at: https://www.mdpi.com/article/10.3390/molecules28217374/s1. Table S1. Formulas of T4 and 25 kinds of OH-PBDEs.

## References

[1] Liu X., Sun L., Wu S., Wang P., Wang Z., Zhai M., Xu J., Zhang D., Yu D., Li C. (2023). Toxicity and risk priority ranking of polybrominated diphenyl ethers (PBDEs): A relative receptor-bound concentration approach. Sci. Total Environ..

[2] Li L., Breivik K. (2019). Global historical stocks and emissions of PBDEs. Environ. Sci. Technol..

[3] Sarkar D., Singh V.K., Singh S.K. (2018). Maternal BDE-209 exposure during lactation perturbs steroidogenesis, germ cell kinetics and THR alpha 1 expression in testes of prepubertal mice offspring. Food Chem. Toxicol..

[4] Liu P., Lu J., Sun J. (2015). Research status of photodegradation of polybromin-ated diphenyl ethers (PBDES). Environ. Chem..

[5] Yao B., Luo Z., Zhi D., Hou D., Luo L., Du S., Zhou Y. (2021). Current progress in degradation and removal methods of polybrominated diphenyl ethers from water and soil: A review. J. Hazard. Mater..

[6] Linares V., Belles M., Domingo J.L. (2015). Human exposure to PBDE and critical evaluation of health hazards. Arch. Toxicol..

[7] Yang Y., Wang L., Zhao Y., Ma F., Lin Z., Liu Y., Dong Z., Chen G., Liu D. (2022). PBDEs disrupt homeostasis maintenance and regeneration of planarians due to DNA damage, proliferation and apoptosis anomaly. Ecotoxicol. Environ. Safe.

[8] Qin W., Li C., Guo L., Ren X., Zhang J. (2019). Binding and activity of polybrominated diphenyl ether sulfates to thyroid hormone transport proteins and nuclear receptors. Environ. Sci.-Proc. Impacts.

[9] Lindqvist D., Wincent E. (2022). Kinetics and toxicity of an environmentally relevant mixture of halogenated organic compounds in zebrafish embryo. Aquat. Toxicol..

[10] Gu C., Cai J., Fan X., Bian Y., Yang X., Xia Q., Sun C., Jiang X. (2020). Theoretical investigation of AhR binding property with relevant structural requirements for AhR-mediated toxicity of polybrominated diphenyl ethers. Chemosphere.

[11] Klaren P.H.M., Geven E.J.W., Nagelkerke A., Flik G. (2012). Kinetics and thiol requirements of iodothyronine 5′-deiodination are tissue-specific in common carp *(Cyprinus carpi* L.). Comp. Biochem. Phys. B.

[12] Vuong A.M., Webster G.M., Romano M.E., Braun J.M., Zoeller R.T., Hoofnagle A.N., Sjoedin A., Yolton K., Lanphear B.P., Chen A. (2015). Maternal polybrominated diphenyl ether (PBDE) exposure and thyroid hormones in maternal and cord sera: The home study, Cincinnati, USA. Environ. Health Perspect..

[13] Zota A.R., Geller R.J., Romano L.E., Coleman-Phox K., Adler N.E., Parry E., Wang M., Park J.-S., Elmi A.F., Laraia B.A. (2018). Association between persistent endocrine-disrupting chemicals (PBDEs, OH-PBDEs, PCBs, and PFASs) and biomarkers of inflammation and cellular aging during pregnancy and postpartum. Environ. Int..

[14] Gustafsson J., Legradi J., Lamoree M.H., Asplund L., Leonards P.E.G. (2023). Metabolite alterations in zebrafish embryos exposed to hydroxylated polybrominated diphenyl ethers. Sci. Total Environ..

[15] Ermilova I., Stenberg S., Lyubartsev A.P. (2017). Quantum chemical and molecular dynamics modelling of hydroxylated polybrominated diphenyl ethers. Phys. Chem. Chem. Phys..

[16] Ou W., Liu H., He J., Yang X. (2018). Development of chicken and fish muscle protein-Water partition coefficients predictive models for ionogenic and neutral organic chemicals. Ecotoxicol. Environ. Safe.

[17] Li F., Xie Q., Li X., Li N., Chi P., Chen J., Wang Z., Hao C. (2010). Hormone activity of hydroxylated polybrominated diphenyl ethers on human thyroid receptor-beta: In vitro and in silico investigations. Environ. Health Perspect..

[18] Ren X.M. (2013). Molecular Toxicology of Hydroxylated Polybrominated Diphenyl Ethers and Perfluoroalkyl Acid on the Thyroid Hormone System.

[19] Ren X.M., Guo L.H. (2012). Assessment of the binding of hydroxylated polybrominated diphenyl ethers to thyroid hormone transport proteins using a site-specific fluorescence probe. Environ. Sci. Technol..

[20] He X. (2015). Study on Interaction Properties of Polybrominated Diphenyl Ethers and Their Derivatives with Biomolecules.

[21] Su Q., Jiang C., Gou D., Long Y. (2021). Surface plasmon-assisted fluorescence enhancing and quenching: From theory to application. ACS Appl. Bio Mater..

[22] Wang L., Liang N., Li H., Yang Y., Zhang D., Liao S., Pan B. (2015). Quantifying the dynamic fluorescence quenching of phenanthrene and ofloxacin by dissolved humic acids. Environ. Pollut..

[23] Paterson K.A., Arlt J., Jones A.C. (2020). Dynamic and static quenching of 2-aminopurine fluorescence by the natural DNA nucleotides in solution. Methods Appl. Fluoresc..

[24] Youssef L., Patra D. (2020). Interaction of carbon nanotubes with curcumin: Effect of temperature and pH on simultaneous static and dynamic fluorescence quenching of curcumin using carbon nanotubes. Luminescence.

[25] Xie Z., Lu G., Qi P. (2014). Effects of BDE-209 and its mixtures with BDE-47 and BDE-99 on multiple biomarkers in Carassius auratus. Environ. Toxicol. Pharmacol..

[26] Cao H., Sun Y., Wang L., Zhao C., Fua J., Zhang A. (2017). Understanding the microscopic binding mechanism of hydroxylated and sulfated polybrominated diphenyl ethers with transthyretin by molecular docking, molecular dynamics simulations and binding free energy calculations. Mol. Biosyst..

[27] Zhao J., Zhu X., Xu T., Yin D. (2015). Structure-dependent activities of polybrominated diphenyl ethers and hydroxylated metabolites on zebrafish retinoic acid receptor. Environ. Sci. Pollut. Res..

[28] Cao L., Zheng Z., Ren X., Andersson P.L., Guo L. (2018). Structure-dependent activity of polybrominated diphenyl ethers and their hydroxylated metabolites on estrogen related receptor gamma: In vitro and in silico study. Environ. Sci. Technol..

