# Supplementary material for: Study on the Biomolecular Competitive Mechanism of Polybrominated Diphenyl Ethers and Their Derivatives on Thyroid Hormones
Source: Molecules. 2023 Oct 31;28(21):7374. doi: 10.3390/molecules28217374 (PMC10650872; doi:10.3390/molecules28217374)
Supplement: Supplementary file 1 [file molecules-28-07374-s001.zip › molecules-2640437-supplementary.pdf]

Table S1: Formulas of T4 and 25 kinds of OH-PBDEs

Serial number	Compounds	Numbers of Br	Formula	Serial number	Compounds	Numbers of Br	Formula
**1**	T_4	0	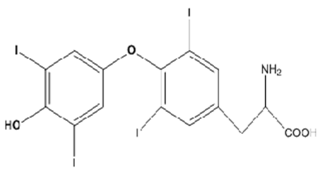 T_4	**14**	4'-OH-BDE-101	5	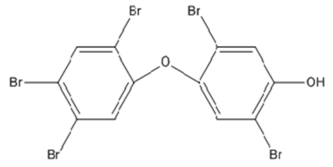 4'-OH-BDE-101
**2**	3'-OH-BDE-7	2	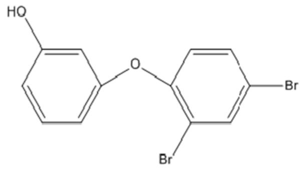 3'-OH-BDE-7	**15**	5'-OH-BDE-99	5	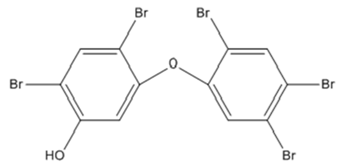 5-OH-BDE-99
**3**	4'-OH-BDE-17	3	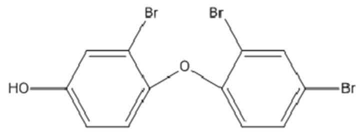 4'-OH-BDE-17	**16**	6'-OH-BDE-99	5	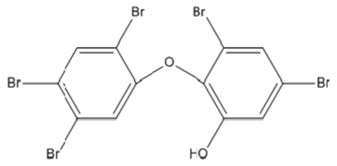 6'-OH-BDE-99
**4**	3'-OH-BDE-28	3	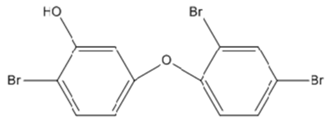 3'-OH-BDE-28	**17**	6-OH-BDE-137	6	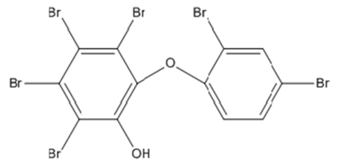 6-OH-BDE-137

Serial number	Compounds	Numbers of Br	Formula	Serial number	Compounds	Numbers of Br	Formula
**5**	2'-OH-BDE-28	3	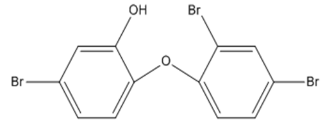 2'-OH-BDE-28	**18**	3'-OH-BDE-154	6	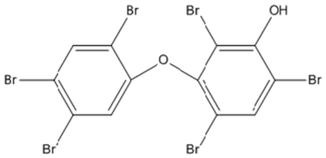 3'-OH-BDE-154
**6**	4-OH-BDE-42	4	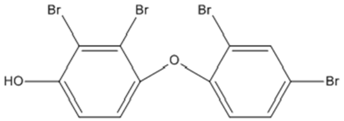 4-OH-BDE-42	**19**	6-OH-BDE-157	6	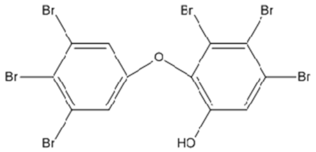 6-OH-BDE-157
7	3-OH-BDE-47	4	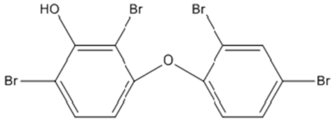 3-OH-BDE-47	20	4-OH-BDE-188	6	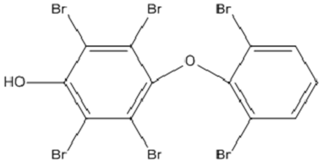 4-OH-BDE-188
8	4'-OH-BDE-49	4	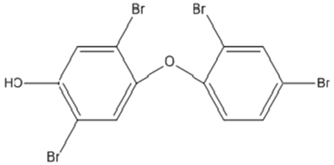 4'-OH-BDE-49	21	6-OH-BDE-180	7	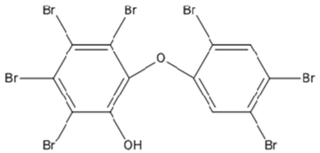 6-OH-BDE-180

Serial number	Compounds	Numbers of Br	Formula	Serial number	Compounds	Numbers of Br	Formula
**9**	6-OH-BDE-47	4	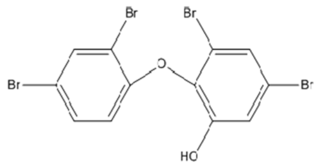 6-OH-BDE-47	**22**	6-OH-BDE-182	7	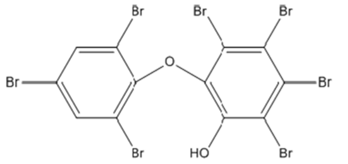 6-OH-BDE-182
**10**	2'-OH-BDE-68	4	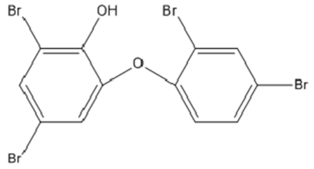 2'-OH-BDE-68	**23**	4'-OH-BDE-201	8	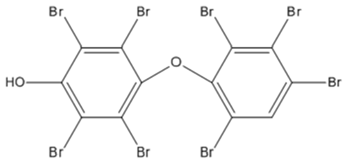 4'-OH-BDE-201
**11**	3-OH-BDE-100	5	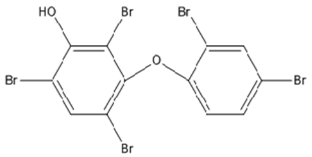 3-OH-BDE-100	**24**	1'-OH-BDE-222	9	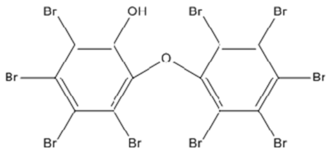 1'-OH-BDE-222
**12**	6-OH-BDE-82	5	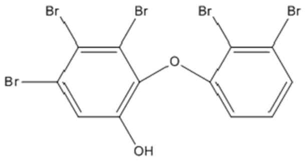 6-OH-BDE-82	**25**	2'-OH-BDE-201	9	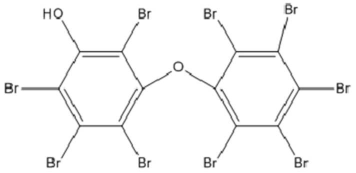 2'-OH-BDE-201

Serial number	Compounds	Numbers of Br	Formula	Serial number	Compounds	Numbers of Br	Formula
**13**	4-OH-BDE-90	5	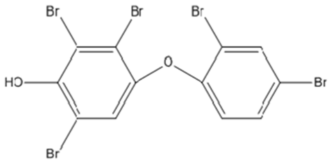 4-OH-BDE-90	**26**	3'-OH-BDE-201	9	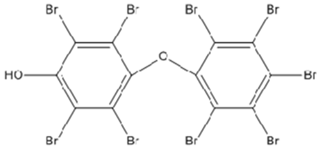 3'-OH-BDE-201
